# Safety of Nurse- and Self-Administered Paediatric Outpatient Parenteral Antimicrobial Therapy

**DOI:** 10.3390/antibiotics9110761

**Published:** 2020-10-30

**Authors:** Shanthy Sriskandarajah, Brett Ritchie, Janet K. Sluggett, Jodie G. Hobbs, Karen J. Reynolds

**Affiliations:** 1 Medical Device Research Institute, College of Science and Engineering, Flinders University, GPO Box 2100, Adelaide, SA 5001, Australia; shanthy.sriskandarajah@flinders.edu.au (S.S.);; 2 Infectious Diseases Department, Women’s and Children’s Hospital, North Adelaide, SA 5006, Australia; Brett.Ritchie@sa.gov.au; 3 UniSA Allied Health and Human Performance, University of South Australia, Adelaide, SA 5000, Australia; janet.sluggett@unisa.edu.au; 4 CPIE Pharmacy Services, Findon, SA 5023, Australia; 5 Centre for Medicine Use and Safety, Faculty of Pharmacy and Pharmaceutical Sciences, Monash University, Parkville, VIC 3052, Australia

**Keywords:** outpatient parenteral antimicrobial therapy, children, safety, adverse events, infusion pumps

## Abstract

This study aimed to compare and contrast the safety and efficacy of nurse- and self-administered paediatric outpatient parenteral antimicrobial therapy (OPAT) models of care and to identify clinical factors associated with documented adverse events (AEs). A total of 100 OPAT episodes among children aged between 1 month and 18 years who were discharged from hospital and who received continuous 24 h intravenous antimicrobial therapy at home via an elastomeric infusion device were included. All documented AEs from the case notes were reviewed by a paediatrician and classified as either major or minor. Multivariable logistic regression was used to determine associations between clinical factors and any AE. A total of 86 patients received 100 treatment OPAT episodes (49 self-administered, 51 nurse administered). The most commonly prescribed antimicrobial via continuous infusion was ceftazidime (25 episodes). Overall, an AE was recorded for 27 (27%) OPAT episodes. Major AEs was recorded for 15 episodes and minor AEs were reported in 14 episodes. The odds of an AE was increased in episodes with self-administration (adjusted odds ratio (aOR) 6.25, 95% confidence interval (CI) 1.44–27.15) and where the duration of vascular access was >14 days (aOR 1.08, 95%CI 1.01–1.15). Our findings suggest minor AEs may be more frequently reported when intravenous antimicrobials are self-administered via 24 h continuous infusions.

## 1. Introduction

Paediatric outpatient parenteral antimicrobial therapy (OPAT) is used as an alternative to hospital inpatient treatment where resources permit. At one Australian paediatric hospital with 334 inpatient beds, the OPAT program is equivalent to 32 beds and is therefore almost 10% of the hospital’s inpatient capacity [1]. Paediatric OPAT has many advantages compared to inpatient treatment including fewer interruptions to family and school life, improved psychological comfort, reduced risk of nosocomial infections and cost savings [1,2,3,4,5,6,7]. The most common OPAT models include antimicrobial administration at a dedicated hospital infusion centre, or administration in the home by the patient or their caregiver (self-administration) or a registered nurse [2,8,9,10]. The OPAT model commonly utilised in the Australian setting for the management of infectious diseases involves continuous 24 h infusions of intravenous antimicrobials in the home setting [11].

Few studies have assessed the safety and efficacy of OPAT in children, with most studies focusing on outcomes associated with adult OPAT services [2]. A recent systematic review comparing hospital with home-based administration of parenteral antimicrobial therapy in children showed that the safety and efficacy of each type of care was similar; however, it was noted that information about the safety of intravenous antimicrobial therapy was scarce [7]. Studies assessing the safety of nurse-administered paediatric OPAT have reported up to 50% of patients experience an adverse event (AE) and up to one quarter of episodes result in hospital readmission [4]. While most OPAT studies evaluate outcomes such as vascular access device (VAD)-related AEs, drug-related AEs and hospital readmission rates, other important outcomes such as infusion-device-related AEs and the frequency of rapid or incomplete infusions are seldom assessed [12].

Evidence from self-administration of OPAT in adults suggests that it is safe and efficacious in carefully selected patients [10,13,14], and this type of care is being increasingly utilised for antimicrobial administration in the home [15]. Cost savings appear to be a significant advantage. The aim of this study was to compare and contrast the safety and efficacy of nurse- and self-administered paediatric OPAT models of care and to identify clinical factors associated with documented AEs.

## 2. Results

A total of 100 treatment episodes (49 self-administered, 51 nurse administered, with no overlaps in categories) among 86 patients were analysed. The mean patient age was 9.13 years (SD 5.12) and 47 (54.7%) were male. Of the 86 patients, 13 received OPAT on two or more separate occasions. Treatment indications included CF (*n* = 44), osteomyelitis (*n* = 17), pneumonia (*n* = 7), septic arthritis (*n* = 4), bacteremia (*n* = 3), primary ciliary dyskinesia (*n* = 3) and others (*n* = 18). The median serum C-reactive protein at hospital admission was 27.5 mg/L (IQR 4.25–130.0) (normal defined as <8.0 mg/L), falling to a median of 6.0 mg/L (IQR 1.0–19.0) by week 3. The median durations are shown in Table 1.

**Figure 1:**
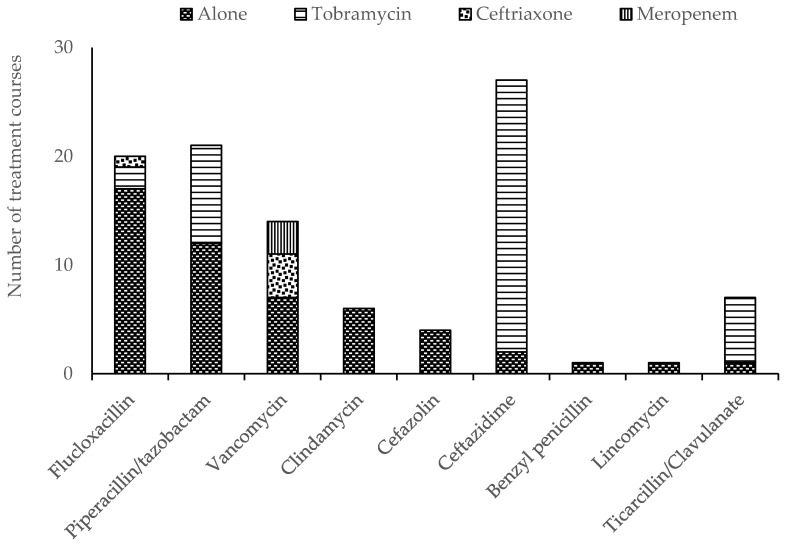
Parenteral antimicrobial therapy prescribed for the administration to children receiving outpatient parenteral antimicrobial therapy (OPAT) in the home.

**Figure 2:**
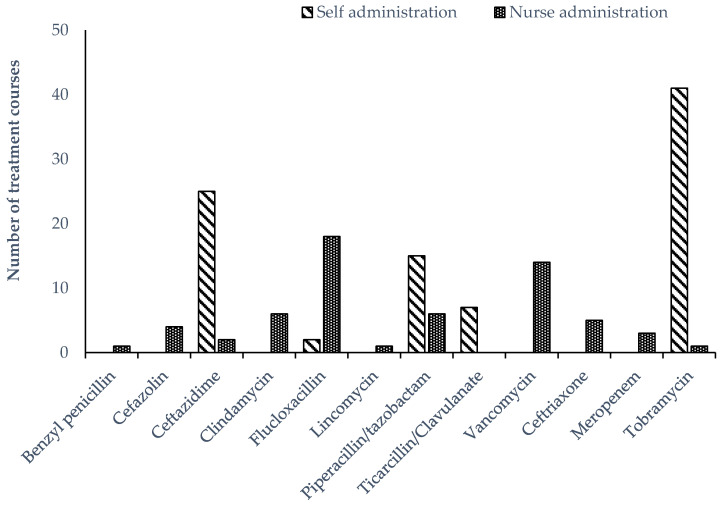
Parenteral antimicrobial therapy prescribed for administration to children receiving OPAT in the home, stratified by nurse or self-administration.

All continuous infusions were delivered using a 24 h elastomeric infusion device (Baxter^®^). A T34L™ syringe driver (Caesaria Medical Electronics) was used to administer parenteral tobramycin. Other intermittent parenteral antimicrobials were administered manually. There were 42 OPAT episodes in the self-administered group and eight episodes in the nurse-administered group where two antimicrobials were prescribed. Tobramycin was the most frequently prescribed antimicrobial overall, given as a single daily dose in combination with a continuous infusion of either ceftazidime or piperacillin/tazobactam (Figure 1). Tobramycin was the most prevalent antimicrobial prescribed in the self-administered group, while flucloxacillin was more frequently prescribed among children receiving nurse-administered OPAT (Figure 2).

Overall, an AE was experienced in one in four OPAT episodes (Table 2). Twice as many AEs were documented in self-administered episodes compared to nurse administered (18 versus 9 episodes). This was primarily due to an increased frequency of minor VAD associated AEs (Table 2). The median time to the first AE was 8 days (IQR 1–14), with further stratification demonstrating a median of 2 days (IQR 1–8) to the first AE among self-administered episodes and 14 days (IQR 5–19) in nurse-administered OPAT. There were more device-related AEs documented in the self-administration group, and more antimicrobial-related AEs documented for patients receiving nurse-administered OPAT.

Associations between clinical factors and any AE explored in the exact logistic regression models are presented in Table 3. There were two variables associated with the greatest odds of the AE: model of antimicrobial administration (self or nurse administration) and vascular access duration (>14 days versus <14 days).

Final results from the multivariable logistic regression model including these two variables showed the odds of experiencing an AE were increased among those with self-administered OPAT (adjusted odds ratio (aOR) 6.25, 95% confidence interval (CI) 1.44–27.15) and those with a vascular access duration of >14 days (aOR 1.08, 95% CI 1.01–1.15) (Table 4).

There were 13 (13%) hospital readmissions. This included eight readmissions from chronic respiratory patients self-administering OPAT (*n* = 49 episodes), of which two readmissions were due to AEs (peripherally inserted central catheter (PICC) line infection and patient cracked PICC line), and the remaining six were due to failure to clinically improve. For the OPAT episodes among patients with non-chronic respiratory infections receiving nurse-administered OPAT (*n* = 51 episodes), there were five readmissions, of which four were due to AEs (red rash over body, medication reaction, fever and rash and PICC line occlusion) and one resulted from failure of the patient to improve. Overall, 89% of all treatment courses were completed as originally prescribed (42 (86%) self-administering, 47 (92%) with nurse-administered OPAT).

## 3. Discussion

Overall, OPAT therapy was successfully completed for the majority of children included in this study, irrespective of infection type. The prevalence of unplanned hospital readmissions was 13% and within the range observed in previous studies (3.8% to 26%) [1,3,4,5,16,17,18]. We identified AEs in more than one quarter of all paediatric OPAT episodes. This finding, while consistent with previous published reports (11% to 50%), [1,3,4,5,16,17,18] indicates that there is a need for prospective investigation and potential improvements across all areas of program delivery. Major AEs were documented in 15% of all episodes and many were potentially preventable. Existing literature suggests factors such as prolonged duration of intravenous therapy, inadequate patient monitoring, line and device complications, and lack of formal medical governance and oversight may be associated with an increased likelihood of AEs during OPAT [16,17].

Closer examination of documented issues relating to infusion devices in this study revealed that two of the five AEs appeared to arise from human errors (incorrect syringe size for the syringe driver and syringe pump incorrectly programmed), and both were in patients receiving care with syringe drivers. Similar errors were also reported by a recent United Kingdom study that assessed incidents arising from syringe drivers in the home [19]. The inability to deliver antibiotic infusion due to failure of the elastomeric balloon device to deflate accounted for two major AEs. Two previous studies assessing the safety of home OPAT for adults in a neighbouring South Australian hospital showed that 23% to 81% of patients had an incomplete infusion documented on at least one occasion [12,20]. We were unable to access case records from the external nursing providers for the present study, which may have led to an underestimation of incomplete infusions, and the true rate of incomplete device emptying may be unrecognised or underreported in patients who are self-administering. This could be investigated prospectively in future studies. We recognize, however, that infrequent and small residual volumes are unlikely to have significant clinical impact on the overall outcomes for children.

In this study, VAD-related AEs were identified in both models of OPAT care. Our findings suggest a need for improved vigilance for VAD safety and monitoring in the home setting, particularly given vascular access line fractures or infection were documented for four OPAT episodes in the self-administered cohort. Conversely, all of the documented antimicrobial-related AEs reported occurred in children receiving nurse-administered OPAT. This may be attributable to a number of factors including differences in the types of patients receiving nurse or self-administered OPAT, the class of antimicrobials received and the side effect profiles of these medications, and the duration of inpatient parenteral antimicrobial therapy prior to home OPAT. Our findings share similarities with a recent prospective Australian study in which the majority of AEs observed in a paediatric OPAT program were related to VADs rather than antimicrobial therapy [1]. The relatively low rate of vascular access line-related infections reported by Hodgson et al. (0.9%) [1] is also consistent with the low rate observed in the present study; however, we observed a greater proportion of unplanned readmissions.

In this study, the time to the first AE in the paediatric home OPAT program was shorter in patients who were self-administering, and self-administration was associated with a six-fold higher odds of any AE compared to nurse administration. However, another study predominately involving adults found no differences in the prevalence of AEs experienced by patients receiving either self- or nurse-administered antimicrobials [14]. It is possible that daily nursing monitoring among paediatric patients receiving home OPAT may not only help to reduce the number of minor AEs, but also delay their onset. However, this hypothesis requires further investigation prospectively given the differences in the duration of hospital inpatient stay and time between VAD placement and discharge between the two patient cohorts, which may explain differences in minor AEs observed in our study.

A strength of this study is the pragmatic comparison of two different models of care provided from a single tertiary centre for children requiring antimicrobial infusions at home. Limitations include the small sample size which limited in-depth exploration of clinical and demographic factors associated with AEs. This is a common limitation of studies assessing the safety and efficacy of paediatric OPAT. In a recent scoping study, 8 of 19 included studies included ≤100 episodes [21]. Our sample size is consistent with a previous study examining the safety and efficacy of OPAT services in adults at another tertiary hospital in Adelaide, South Australia [13]. In addition, our sample was similar to other studies in terms of patient age and main infections [16,21]. Other limitations include the difference in patient cohorts, the retrospective nature of the study and possible differences in AE recording and/or reporting between the two patient cohorts. It is possible that minor AEs were not documented in case notes and patients who were self-administering may not recognise or mention minor AEs at their clinic visit. The inability to access case notes from the external nursing provider who provided OPAT services in the nurse-administered group means that minor AEs among the nurse-administered group may have been further underestimated. Some of the disadvantages of OPAT that could not be assessed in the present study include potential communication difficulties between patient/carers and health providers, patient/caregiver concern relating to the potential for complications including after-hours emergencies, and lack of comprehensive services such as multi-disciplinary care [22].

Opportunities for improved delivery of our paediatric OPAT program identified through this audit may also be applicable to other similar OPAT settings. Firstly, and most importantly, our results are consistent with previous studies that infer greater emphasis should be given to VAD care and mitigating where possible the risks associated with these devices in the home setting. In our current OPAT model of care routine monitoring, estimating and documenting of the volume of antibiotic infusion delivered via the elastomeric balloon device during a 24 h period is not performed. Additionally, re-evaluation of the existing ambulatory infusion devices within our OPAT service is required to ensure that we have the optimal type and design. We were unable to quantify the extent to which the underreporting of AEs and other outcomes occurred, and this could be explored prospectively, along with staff knowledge and practices. Finally, requesting written feedback from patients and their informal caregivers following an OPAT episode may identify further quality improvement opportunities. These elements are in keeping with existing good practice recommendations for paediatric OPAT services [2].

## 4. Materials and Methods

### 4.1. Population and Setting

The Women’s and Children’s Hospital (WCH) is the major metropolitan children’s teaching hospital in Adelaide, South Australia, with over 200 paediatric beds. All eligible patients are assessed by a paediatrician and/or the infectious diseases specialist for their suitability for OPAT prior to discharge. Patient eligibility criteria for OPAT include: (1) aged between 1 month and 18 years, (2) presence of a PICC or central VAD, (3) part or all of the antimicrobial therapy delivered in the home, (4) patient has family and/or carer home supports and skills to supervise and deliver care, (5) clinically stable or improving and without significant or serious comorbidity, (6) patient can be reviewed by medical staff weekly or more frequently if required, and (7) patient resides in metropolitan Adelaide.

At the time of this audit, the WCH provided two models of OPAT services to paediatric patients: self-administration (by the parent, legal caregiver or adolescent patient if appropriate), and nurse administration. We used this unique opportunity to contrast the differences and similarities between these models of care delivered from a single hospital.

Children with chronic respiratory diseases (e.g., cystic fibrosis (CF), primary ciliary dyskinesia and chronic bronchiectasis) under the care of a respiratory physician at the WCH and who require prolonged antimicrobial treatment are eligible for self-administered OPAT. At this hospital, the respiratory nurse provides education and training on self-administration to either the parent or legal carer, or the adolescent, who must demonstrate satisfactory competency to deliver parenteral antimicrobial therapy safely and independently before discharge. All syringe drivers were preprogramed by the nurses only, prior to discharge, with no patient self-programming. The parent and/or carer is also instructed to contact the respiratory OPAT nurse or on-call respiratory paediatrician for advice and assistance if any problems emerge during treatment. Any AEs reported by the patient, parent and/or carer are recorded by the respiratory OPAT nurse or on-call paediatrician in the patient records maintained by the hospital.

Children diagnosed with conditions such as complicated osteoarticular infections, cerebral infections, bacterial endocarditis and severe complicated pneumonia with empyema and require OPAT are referred to the Metropolitan Referral Unit (MRU). This model of care engages nursing services from an external community-based nursing service. After the patient returns home, a nurse will visit each day to replace the antimicrobial infusion device and/or administer parenteral antimicrobials, check the patient’s vascular access, change the vascular access dressing if required and monitor the clinical progress of the patient. If any problems arise, the nursing provider contacts the appropriate hospital unit directly for specific advice and/or assistance. The nurse is also trained to record any AEs into the patient records maintained by the external provider. After completion of the prescribed OPAT course, the nursing provider sends a written discharge letter to the WCH via the MRU, which is filed in the patients’ WCH medical records. In both models of care, all patients receive a weekly medical review at the WCH.

### 4.2. Study Design and Consent

A retrospective audit of 100 OPAT episodes among children discharged from the WCH between January 2013 and June 2017 was undertaken. Treatment episodes were first identified using the WCH pharmacy database that captures details of all parenteral infusions supplied for OPAT. Only OPAT patients prescribed continuous, 24 h parenteral antimicrobial therapy via an ambulatory elastomeric infusion device and with a PICC or midline catheter inserted were included in this study. Patients with an infusaport^®^ and those living in a rural or remote location were excluded. Patient case notes were requested from the WCH medical records department. The first 100 consecutive OPAT patient records received were reviewed. Ethics approval was obtained from the WCH Human Research Ethics Committee (ID number 887A/September/2019). All eligible patients/families were posted information about the audit and were given the opportunity to opt out prior to audit commencement.

### 4.3. Data Collection

Patient demographic data, primary medical diagnoses, laboratory investigations and details of parenteral antimicrobial therapy were collected for each OPAT course. Data were collected from the hospital’s patient medical records and electronic clinical information system, and from the external nursing provider’s discharge summaries for patients who received nurse-administered OPAT. Patients were followed until final discharge from the OPAT service. Outcome data collected included: (1) completion of treatment as prescribed by the referring paediatrician, (2) unplanned or unscheduled readmission to hospital, and (3) all documented AEs related to the VAD, infusion device or antimicrobials. Data were collected using a Microsoft^®^ Excel 2016 (Washington, DC, USA)—based data collection tool, which was piloted, used in a related study in adults [12], and then refined prior to study commencement.

### 4.4. Criteria Used to Define Adverse Events

Details of all documented AEs were reviewed by an infectious disease paediatrician and pharmacist, and classified as either major or minor with discrepancies resolved after discussion. AEs requiring minimal clinical intervention (e.g., treatment for PICC line leakage or ooze at the insertion site not requiring repair or replacement, mild bruising or bleeding, local pruritus) were considered to be minor AEs [12]. A major AE was considered to be any event requiring greater than minimal medical and/or nursing intervention, including a change or cessation of antimicrobial therapy, unexpected replacement of a VAD or infusion device, and/or unplanned readmission to hospital. Major AEs were further classified as VAD, infusion device or antimicrobial-related events. Major AEs relating to VADs included temporary or permanent line occlusion from thrombosis or other cause, line-related infections, and line fractures and/or breakages [12,14]. Any AEs relating to the infusion device, including failure of the elastomeric balloon to deflate, leaking or other mechanical failure were considered to be a major event [12]. Major AEs relating to antimicrobials included any documented medication-related renal impairment (≥10% decrease in creatinine clearance from baseline), hepatic impairment (defined as an increase in hepatic enzymes ≥3 times the upper limit of normal), thrombocytopenia (platelet count <150 × 109/L), neutropenia (<1.0 × 109/L) or eosinophilia (≥0.5 × 109/L) [5,12,20]. In addition, any clinically significant AEs relating specifically to antimicrobial therapy that required intervention were reported [23].

### 4.5. Sample Size Justification

Based on the number of patients admitted to the service annually (approximately 70–80 patients per year) and available project resources, we selected a convenience sample of 100 episodes of care to examine the safety and efficacy of paediatric OPAT.

### 4.6. Statistical Analysis

Data were transferred from Microsoft Excel^®^ to SPSS (version 25) for analysis. Continuous data were summarised using means and standard deviations (SDs) and skewed data were summarised using medians and interquartile ranges (IQRs).

The following covariates were eligible for inclusion in the multivariable analysis: number of antimicrobials (categorised as one or more than one), model of antimicrobial administration (self or nurse administration), number of concurrent infusion devices utilised by the patient (one device or more than one), vascular access duration (short vs. long, i.e., ≤14 versus >14 days) and total antimicrobial treatment days (short vs. long, i.e., ≤14 versus >14 days) based on findings of previous studies [16,24,25]. The 14 day cut-point used to define short and long therapy was selected as the median duration of vascular access and total antimicrobial treatment days were both 14 days.

When the ratio of the numbers of AEs per covariate analysed is less than 10, the validity of the logistic model becomes problematic [26]. For this reason, we first examined unadjusted (bivariate) associations between the five covariates of interest and the outcome of any AE using exact logistic regression. However, inclusion of all five covariates in a multivariable exact logistic model demonstrated collinearity with two sets of variables: (1) vascular access duration and total antimicrobial treatment days, and (2) the number of infusion devices utilised and the model of antimicrobial administration. Therefore, to identify the covariates that best explained the likelihood of an AE, we then used exact logistic regression to further examine adjusted associations in five different models that each included three covariates. Based on these findings, we were able to utilise multivariable logistic regression in our final analysis to examine associations between the two clinical factors associated with the greatest odds of the AE. Regression analyses were restricted to the first OPAT episode for each patient (*n* = 86 episodes). *p*-values < 0.05 were considered statistically significant.

## 5. Conclusions

Overall, an AE was recorded for just over one quarter of all paediatric OPAT episodes. Administration of antimicrobials to patients with chronic respiratory disease by using a self-administration model of care in the home setting may be associated with early and frequent minor AEs; however, differences in the patient treatment indications, models of care, duration of hospital inpatient stay and duration of VAD placement prior to hospital discharge limit our ability to draw conclusions regarding differences between the two models of care. Until we can conclusively demonstrate no significant difference between the two OPAT models, using similar patient types studied prospectively, our preference is for a nurse administered and supervised OPAT model of care in the paediatric setting. However, this must be balanced with other relevant factors including convenience, cost and patient preferences, knowledge and skills.

## Figures and Tables

**Table 1:** Median duration (interquartile range) of treatment.

Clinical Factors	Overall *n* = 100	Self-Administered *n* = 49	Nurse Administered *n* = 51
OPAT duration (days)	11.0 (8.0–14.0)	11.0 (10.0–12.0)	12.0 (7.0–24.0)
Duration of hospital inpatient stay (days)	6 (2.75–9.0)	2.0 (2.0–4.0)	9.0 (7.0–14.0)
Duration the vascular access line was in place (days)	14.0 (13.25–24.0)	14.0 (13.5–14.0)	24.0 (13.0–35.0)
Duration of antimicrobial treatment (hospital stay + OPAT) (days)	14.5 (14.5–24.75)	14.0 (14.0–14.0)	24.0 (15.0–32.0)

OPAT—outpatient parenteral antimicrobial therapy.

**Table 2:** Major and minor adverse events documented among children receiving OPAT.

OPAT Episodes	Total *n* = 100	Self-Administered *n* = 49	Nurse Administered *n* = 51
OPAT episodes with any adverse event (%) ^a^	27 (27.0)	18 (36.7)	9 (17.6)
Minor adverse event (%)	14 (14.0)	12 (24.5)	2 (3.9)
Major adverse event (%)	15 (15.0)	7 (14.3)	8 (15.7)
OPAT episodes with a VAD-related adverse event (%)	21 (21.0)	16 (32.7)	5 (9.8)
Minor adverse event (%) ^b^	14 (14.0)	12 (24.5)	2 (3.9)
Dermatitis/erythema/pruritus/mild blistering	7	6	1
Kink in line (transient)	3	2	1
Haematoma/blood ooze/leakage from PICC site	4	4	0
Major adverse event (%)	8 (8.0)	4 (8.2)	4 (7.8)
PICC line infection	1	1	0
PICC line occlusion	2	0	2
Fractured PICC requiring replacement	3	3	0
Patient removed line	1	0	1
Allergic reaction to VAD dressing	1	0	1
OPAT episodes with an infusion-device-related adverse event (%)	4 (4.0)	4 (8.2)	0 (0.0)
Minor adverse event (%)	0 (0.0)	0 (0.0)	0 (0.0)
Major adverse event (%)	4 (4.0)	4 (8.2)	0 (0.0)
Incorrect syringe size for syringe driver (tobramycin)	1	1	0
Syringe pump occlusion (tobramycin)	1	1	0
Syringe pump incorrectly programmed	1	1	0
Failure of elastomeric balloon to deflate	2	2	0
OPAT episodes with an antimicrobial-related adverse event (%)	4 (4.0)	0 (0.0)	4 (7.8)
Minor adverse event (%)	0 (0.0)	0 (0.0)	0 (0.0)
Major adverse event (%)	4 (4.0)	0 (0.0)	4 (7.8)
Rash	3	0	3
Raised liver function tests	1	0	1

OPAT—outpatient parenteral antimicrobial therapy; PICC—peripherally inserted central catheter; VAD—vascular access device. ^a^ There were 25 OPAT episodes with 1 adverse event recorded and 2 episodes with 2 adverse events recorded. ^b^ includes skin erythema, pruritus, minor blistering, rash, hematoma, minor leakage at the insertion site.

**Table 3:** Results of bivariate and multivariate exact logistic regression models to examined clinical factors associated with an adverse event (*n* = 86 OPAT episodes).

Clinical Factors	Bivariate Regression Models (1 Covariate) Unadjusted OR (95% CI)	Multivariable Model (all 5 Covariates) Adjusted OR (95% CI)	Model 1 (3 Covariates) Adjusted OR (95% CI)	Model 2 (3 Covariates) Adjusted OR (95% CI)	Model 3 (3 Covariates) Adjusted OR (95% CI)	Model 4 (3 Covariates) Adjusted OR (95% CI)
Treated with 2 or more antimicrobials (ref: 1 antimicrobial)	1.75 (0.58–5.43)	4.05 (0.44–35.93)	0.99 (0.20–4.61)	0.96 (0.20–4.28)	1.96 (0.26–11.66)	2.31 (0.30–14.77)
Self-administration (ref: nurse administration)	2.40 (0.80–7.56)	16.91 (1.22–410.27) *	5.23 (0.86–44.01)	2.57 (0.43–16.04)		
Using 2 or more infusion devices concurrently (ref: using 1 device)	1.46 (0.47–4.46)	0.05 (0.00–1.41)			1.15 (0.17–9.96)	0.55 (0.06–5.74)
Vascular access duration >14 days (ref: ≤14 days)	1.45 (0.48–4.40)	12.94 (1.28–723.72) *	3.90 (0.87–24.79)		1.96 (0.55–7.63)	
Antimicrobial treatment duration > 14 days (ref: ≤14 days)	0.65 (0.21–1.95)	0.15 (0.00–1.70)		1.17 (0.29–5.15)		0.62 (0.15–2.57)

* Inflated confidence intervals. CI: confidence interval, OR: odds ratio.

**Table 4:** Adjusted associations between clinical factors and any adverse event (*n* = 86 episodes) *.

Clinical Factors	Adjusted Odds Ratio (95% Confidence Interval)	*p*-Value
Self-administration (ref: nurse administration)	6.25 (1.44–27.15)	0.014
Vascular access duration >14 days (ref: ≤14 days)	1.08 (1.01–1.15)	0.028

* Multivariable logistic regression model.

## References

[1] Hodgson K.A., Huynh J., Ibrahim L.F., Sacks B., Golshevsky D., Layley M., Spagnolo M., Raymundo C.-M., Bryant P.A. (2016). The use, appropriateness and outcomes of outpatient parenteral antimicrobial therapy. Arch. Dis. Child..

[2] Patel S., Abrahamson E., Goldring S., Green H., Wickens H., Laundy M. (2014). Good practice recommendations for paediatric outpatient parenteral antibiotic therapy (p-OPAT) in the UK: A consensus statement. J. Antimicrob. Chemother..

[3] Akar A., Singh N., Hyun D.Y. (2014). Appropriateness and safety of outpatient parenteral antimicrobial therapy in children: Opportunities for pediatric antimicrobial stewardship. Clin. Pediatr.

[4] Gomez M., Maraqa N., Alvarez A., Rathore M. (2001). Complications of outpatient parenteral antibiotic therapy in childhood. Pediatr. Infect. Dis. J..

[5] Madigan T., Banerjee R. (2013). Characteristics and Outcomes of Outpatient Parenteral Antimicrobial Therapy at an Academic Children’s Hospital. Pediatr. Infect. Dis. J..

[6] Rathore M.H. (2010). The Unique Issues of Outpatient Parenteral Antimicrobial Therapy in Children and Adolescents. Clin. Infect. Dis..

[7] Bryant P.A., Katz N.T. (2018). Inpatient versus outpatient parenteral antibiotic therapy at home for acute infections in children: A systematic review. Lancet Infect. Dis..

[8] Tice A.D., Rehm S.J., Dalovisio J.R., Bradley J.S., Martinelli L.P., Graham D.R., Gainer R.B., Kunkel M.J., Yancey R.W., Williams D.N. (2004). Practice guidelines for outpatient parenteral antimicrobial therapy. Clin. Infect. Dis..

[9] Maraqa N., Rathore M.H. (2010). Pediatric Outpatient Parenteral Antimicrobial Therapy: An Update. Adv. Pediatr..

[10] Kieran J., O’Reilly A., Parker J., Clarke S., Bergin C. (2009). Self-administered outpatient parenteral antimicrobial therapy: A report of three years experience in the Irish healthcare setting. Eur. J. Clin. Microbiol. Infect. Dis..

[11] Sluggett J.K., Ritchie B., Lombardo G., Sluggett A.J. (2020). Continuous Intravenous Antibiotic Infusions in the Home Site of Care: Practical Tips for Providers. Infusion.

[12] Sriskandarajah S., Ritchie B., Eaton V., Sluggett J.K., Hobbs J.G., Daniel S., Reynolds K.J. (2020). Safety and Clinical Outcomes of Hospital in the Home. J. Patient Saf..

[13] Matthews P.C., Conlon C.P., Berendt A., Kayley J., Jefferies L., Atkins B., Byren I. (2007). Outpatient parenteral antimicrobial therapy (OPAT): Is it safe for selected patients to self-administer at home? A retrospective analysis of a large cohort over 13 years. J. Antimicrob. Chemother..

[14] Subedi S., Looke D., McDougall D., Sehu M., Playford E. (2015). Supervised self-administration of outpatient parenteral antibiotic therapy: A report from a large tertiary hospital in Australia. Int. J. Infect. Dis..

[15] Barr D., Semple L., Seaton R. (2012). Outpatient parenteral antimicrobial therapy (OPAT) in a teaching hospital-based practice: A retrospective cohort study describing experience and evolution over 10 years. Int. J. Antimicrob. Agents.

[16] Le J., Agustin M.S., Hernandez E.A., Tran T.T., Adler-Shohet F.C. (2010). Complications Associated With Outpatient Parenteral Antibiotic Therapy in Children. Clin. Pediatr..

[17] Mace A.O., McLeod C., Yeoh D.K., Vine J., Chen Y.-P., Martin A.C., Blyth C.C., Bowen A.C. (2017). Dedicated paediatric Outpatient Parenteral Antimicrobial Therapy medical support: A pre–post observational study. Arch. Dis. Child..

[18] Sriskandarajah S., Hobbs J.G., Roughead E.E., Ryan M., Reynolds K.J. (2018). Safety and effectiveness of ‘hospital in the home’ and ‘outpatient parenteral antimicrobial therapy’ in different age groups: A systematic review of observational studies. Int. J. Clin. Pr..

[19] Lyons I., Blandford A. (2018). Safer healthcare at home: Detecting, correcting and learning from incidents involving infusion devices. Appl. Ergon..

[20] Pandya K.H., Eaton V., Kowalski S., Sluggett J.K. (2017). Safety of continuous antibiotic infusions administered through an Australian hospital in the home service: A pilot study. J. Pharm. Pr. Res..

[21] Carter B., Carrol E.D., Porter D., Peak M., Taylor-Robinson D., Fisher-Smith D., Blake L. (2018). Delivery, setting and outcomes of paediatric Outpatient Parenteral Antimicrobial Therapy (OPAT): A scoping review. BMJ Open.

[22] Minton J., Murray C.C., Meads D., Hess S., Vargas-Palacios A., Mitchell E., Wright J., Hulme C., Raynor D.K., Gregson A. (2017). The Community IntraVenous Antibiotic Study (CIVAS): A mixed-methods evaluation of patient preferences for and cost-effectiveness of different service models for delivering outpatient parenteral antimicrobial therapy. Health Serv. Deliv. Res..

[23] Hoffman-Terry M., Fraimow H., Fox T., Swift B., Wolf J. (1999). Adverse effects of outpatient parenteral antibiotic therapy. Am. J. Med..

[24] Montalto M., Lui B., Mullins A., Woodmason K. (2010). Medically-managed Hospital in the Home: 7 year study of mortality and unplanned interruption. Aust. Health Rev..

[25] Seaton R., Sharp E., Bezlyak V., Weir C. (2011). Factors associated with outcome and duration of therapy in outpatient parenteral antibiotic therapy (OPAT) patients with skin and soft-tissue infections. Int. J. Antimicrob. Agents.

[26] Peduzzi P., Concato J., Kemper E., Holford T.R., Feinstein A.R. (1996). A simulation study of the number of events per variable in logistic regression analysis. J. Clin. Epidemiol..

